# The influence of climate warming on flowering phenology in relation to historical annual and seasonal temperatures and plant functional traits

**DOI:** 10.7717/peerj.15188

**Published:** 2023-04-21

**Authors:** Cole Geissler, Allison Davidson, Richard A. Niesenbaum

**Affiliations:** 1 Department of Biology, Muhlenberg College, Allentown, PA, United States of America; 2 Department of Mathematics, Muhlenberg College, Allentown, PA, United States of America

**Keywords:** Climate change, Phenology, Herbarium specimens, Seasonal temperatures, Plant functional characteristics

## Abstract

Climate warming has the potential to influence plant flowering phenology which in turn can have broader ecological consequences. Herbarium collections offer a source of historical plant data that makes possible the ability to document and better understand how warming climate can influence long-term shifts in flowering phenology. We examined the influence of annual, winter, and spring temperatures on the flowering phenology of herbarium specimens for 36 species collected from 1884–2015. We then compared the response to warming between native and non-native, woody and herbaceous, dry and fleshy fruit, and spring vs summer blooming species. Across all species, plants flowered 2.26 days earlier per 1 °C increase in annual average temperatures and 2.93 days earlier per 1 °C increase in spring onset average temperatures. Winter temperatures did not significantly influence flowering phenology. The relationship of temperature and flowering phenology was not significantly different between native and non-native species. Woody species flowered earlier than herbaceous species only in response to increasing annual temperatures. There was no difference in the phenological response between species with dry fruits and those fleshy fruits for any of the temperature periods. Spring blooming species exhibited a significantly greater phenological response to warming yearly average temperatures than summer blooming species. Although herbarium specimens can reveal climate change impacts on phenology, it is also evident that the phenological responses to warming vary greatly among species due to differences in functional traits such as those considered here, as well as other factors.

## Introduction

Since the late 19th century the average global temperature has risen by 1.1 °C (IPCC, 2021), and in the contiguous United States, the yearly average temperature has increased by 0.09 °C each decade since 1895, surpassing the 1895–2020 average in 1956 (NOAA, 2021). In the region in which this study was conducted, the yearly average temperature increased by 0.11 °C each decade since 1895, surpassing the 1895–2021 average in 1958 (NOAA, 2022). This change in climate has the potential to influence many biological and ecological processes, particularly changes in phenology (timing of biological events) in plants and animals, given the potential for temperature-dependence of such traits (Walther et al., 2002). Moreover, the effects of climate change on phenology may in turn have further implications in terms of the consequential impact on ecological interactions. Among these is the potential impact of climate change on pollination due to the differential temporal response of plants and their pollinators to warming seasonal temperatures resulting in asynchrony between flowering time and the emergence of pollinators (Liu et al., 2011; Rafferty & Ives, 2012; Robbirt et al., 2014; Forrest, 2015; Rafferty, 2017; Renner & Zohner, 2018). Similarly, a differential response to warming between prey emergence and predator demand has been shown to significantly impact different trophic levels in the same community (Harrington, Woiwod & Sparks, 1999). Additionally, independent shifts in the phenology of invasive plants and their herbivores in response to warming suggest that climate change will influence biological invasions (Walther et al., 2002; Walther et al., 2009; Hellmann et al., 2008).

Given the potential for climate warming to influence plant flowering phenology and the broader ecological consequences of such changes, this has become an important area of study. The effect of warming on flowering phenology has been addressed experimentally through the physical warming of plots in natural settings (Price & Waser, 1998; Memmott et al., 2007; Flynn & Wolkovich, 2018; Zettlemoyer, Schultheis & Lau, 2019; Dorji et al., 2020; Pérez-Ramos et al., 2020; Collins et al., 2021); in field studies over temperature gradients with elevation (Hoiss, Krauss & Steffan-Dewenter, 2015; Inouye, 2020; Richman et al., 2020) and latitude (Parmesan, 2007; Bertin, 2008; Niesenbaum, 2012; De Manincor et al., 2020), and along urban-rural temperature gradients resulting from heat island effects in cities (Neil & Wu, 2006). Another approach has been to use historical data from herbarium collections to assess the effects of warming on flowering phenology. Herbarium specimens are most often collected during blooming, indicating flowering dates and location, and many such collections span hundreds of years, offering a unique long-term historical perspective of the relationship between flowering time and historical trends in warming (Meineke, Davis & Davies, 2018).

The potential limitations of using herbarium specimens to document long-term changes in flowering time stem from collection biases due to non-random collecting practices related to location, sampling time, and collector preferences for certain species, as well as duplicate sampling of individual species from the same location on the same date (Robbirt et al., 2011; Daru et al., 2018; Willis et al., 2017; Gamble & Mazer, 2022; Yang et al., 2022). Although these biases can limit the value of phenological information of individual specimens, the use of large sample sizes while avoiding duplicates; and including plants from a large number of families, and with different functional traits and flowering seasons can allow for a more accurate assessment of the long-term shifts in phenology at a given location (Willis et al., 2017; Daru et al., 2018). Bias can also come from inconsistent assessment of phenophase or the timing of onset, peak, and end flowering dates, which becomes more problematic for species that flower over longer parts of a season. This can be mitigated by using a consistent application of flowering time based on the percentage of open flowers, and by using plants with similar durations of flowering (Davis et al., 2015). Finally, the use of the collection date of flowering herbarium specimens as a proxy for actual flowering time has been validated in studies that show no significant difference between specimen collection dates and flowering dates observed in the field in the same year (Bolmgren & Lönnberg, 2005; Miller-Rushing et al., 2006; Robbirt et al., 2011; Jones & Daehler, 2018; Ramirez-Parada, Park & Mazer , 2022; Bloom, O’Leary & Riginos, 2022).

Prior studies on long-term trends in flowering phenology in relation to historical temperatures revealed changes in flowering time by as much as 2–6 days earlier per 1 °C increase in temperature (Primack et al., 2004; Panchen et al., 2012; Park & Schwartz, 2015; Davis et al., 2015), and 8–16 days earlier per 1 °C in more recent decades (Primack et al., 2004; Lavoie & Lachance, 2006). However, not all species show the same temporal response, with some blooming earlier and others later over time (McEwan et al., 2011; Calinger, Queenborough & Curtis, 2013; Hart et al., 2014; Davis et al., 2015; Bertin et al., 2017; Panchen & Gorelick, 2017). One possible reason for observed interspecific differences in response to warming is that individual species may respond differently to seasonal temperatures within a year, such that winter or spring temperatures could independently or divergently influence flowering time depending on species specific cues for germination, and leaf or flower emergence (Cook, Wolkovich & Parmesan, 2012; Pearson, 2019). Many prior studies of climate warming on flowering phenology have been based solely on the temporal trends using collection year as the only explanatory variable. While this may be well suited for examining long term trends in phenology with warming, since temperatures have risen during the collection periods; linking collection dates to concurrent temperature data from the same locations allows for the consideration of seasonal temperatures within a year as drivers of phenological change. Use of temperature data can also more accurately reveal year-to-year variation in phenological response to temperature (Holle, Wei & Nickerson, 2010; Panchen et al., 2012; Bertin, 2015; Davis et al., 2015; Park & Schwartz, 2015; Daru et al., 2018; Ramirez-Parada, Park & Mazer , 2022; Zettlemoyer, Wilson & De Marche, 2022).

Another reason for mixed results among species in prior studies of warming on flowering phenology may be due to differences in plant functional characteristics known to be related to phenology (Parmesan & Hanley, 2015). Interspecific character differences that likely influence temporal responses to warming include native status (native *versus* non-native species), growth habit (woody *versus* herbaceous species), fruit type (species with dry *versus* fleshy fruit), and seasonality of blooming (spring-blooming *versus* summer-blooming species). The native status of species could influence phenological responses to temporal warming because non-native species, especially those that are invasive, tend to exhibit more phenotypic plasticity than native species (Richards et al., 2006; Davidson, Jennions & Nicotra, 2011), and thus may be more likely to exhibit changes in flowering time in response to warming temperatures especially over the short term (Calinger, Queenborough & Curtis, 2013; Wolkovich et al., 2013; Zettlemoyer, Schultheis & Lau, 2019). Growth habit also has the potential to influence the temporal changes in flowering time. There is some evidence that in woody species phenology may tend to be cued by photoperiod more than temperature, so woody species may be less likely to exhibit changes in flowering time with temperature deviations (Saikkonen et al., 2012; Zohner et al., 2016; Flynn & Wolkovich, 2018). Fruit type may influence the phenological response to warming since plants with fleshy fruits may be more constrained by fruit and seed development time and disperser abundance than timing for pollination (Primack, 1987; Du et al., 2020), which in turn could influence the response to warming. Also, accounting for the season in which different species bloom can shed further light on interspecific divergent responses to warming (Pearson, 2019).

The objective of this study was to use herbarium specimens for 36 species from 28 families collected from 1884–2015 to determine general patterns of long-term changes in flowering time in relation to concurrent historical temperature data in eastern Pennsylvania, USA. We also assessed whether yearly average temperature, and winter and spring temperatures elicit different responses across species. Additionally, our goal was to determine the extent to which differences in native status, growth habit, fruit type, and the seasonality of blooming are related to the response of flowering phenology to warming. We predicted that flowering time will tend to occur earlier in response to warming over the time period considered, and that there would be differences among species in the predictive strength of yearly average, and winter and spring temperatures on flowering phenology trends. We also hypothesized that the relationship between flowering phenology and warming temperatures would be stronger in non-native species in contrast to native species, in herbaceous compared to woody species, in species with dry fruits relative to those with fleshy fruits, and in spring blooming species compared to those that flower in summer.

## Methods

### Region of study

We studied historical flowering times in relation to temperature using herbarium specimens from Berks, Bucks, Lehigh, and Northampton counties in the Lehigh and Delaware River watersheds in eastern Pennsylvania. This area is characterized by fragmented mature deciduous forest nested within a largely agricultural, suburban matrix. Typical of oak-hickory, mesophytic forests in this region, the dominant tree species are *Quercus* and *Carya* spp., *Acer rubrum*, *Fagus grandifolia*, *Liriodendron tulipifera*, and *Juglans nigra* (Braun, 1950; Dyer, 2006). Development in these areas has been restricted primarily to agricultural land along the urban corridor within the study region with the greatest suburban expansion occurring from the mid-1990s to the present (Lehigh Valley Planning Commission, 2022). The growing season in this region averages 177 days with the last frost occurring in early April and the first frost in mid-October (Halma & Oplinger, 2001).

### Climate data

We obtained historical climate data from the National Oceanic and Atmospheric Administration (NOAA) through the Climate Data Online service and Global Summary of the Month CSV datasets (Lawrimore et al., 2016). To best represent temperatures across the region over time, and because data from individual weather stations are often incomplete, we collected the daily average temperatures between 1884 and 2015 from 11 individual climate stations located in the study area with the most extensive historical coverage. Then, because of minimal differences in temperatures among stations, we averaged the daily temperature data values across all climate stations in the four counties. We then calculated the monthly averages of the daily temperatures (TAVG) across the counties in our study, and used them to calculate average annual temperatures (YearTAVG). We calculated winter temperatures for each year as an average of the December (prior year), January and February temperatures (WinterTAVG), and the average spring onset temperatures for each year as an average of March, April, and May temperatures (SpringTAVG) to determine the extent to which these seasonal temperatures are related to phenological change (Primack et al., 2004; Bertin, 2015; Davis et al., 2015). Although we recognize that the lack of continuity of temperature data across weather stations could potentially be problematic in that different stations contribute data in different years, we minimized this potential by using a large number of weather stations with overlapping data and by averaging across stations on any given date. This allowed for a more accurate representation of annual variation in phenological response to temperature and the consideration of seasonal temperatures within a year as drivers of phenological change (Holle, Wei & Nickerson, 2010; Panchen et al., 2012; Bertin, 2015; Davis et al., 2015; Park & Schwartz, 2015; Daru et al., 2018).

### Phenology data

We obtained historical flowering times using physical and digital herbarium specimens from the herbaria at Muhlenberg College, The Academy of Natural Sciences, and The Morris Arboretum. We sourced digital specimens from the Mid-Atlantic Megalopolis Project through the Mid-Atlantic Herbaria Consortium. We selected insect-pollinated species from these collections that had at least 30 specimens collected in our study area at peak flowering after the removal of duplicate flowering specimens with identical date and location. Peak flowering was determined as the presence of buds, flowers and fruits with ≥75% fully opened flowers (Davis et al., 2015). We excluded herbarium specimens from before 1884 because of limited climate data for that time period, and after 2015 due to reduced collection rates. None of the specimens used were collected from large urban centers or industrial sites. Based on the above criteria, we were able to include 36 species with large enough sample sizes across our categories of interest (native status, growth form, fruit type, and blooming season (Table 1).

**Table 1:** Study species and their categorical classifications.

Species	Family	Native status	Growth type	Fruit type	Seasonality
*Amelanchier arborea*	Rosaceae	Native	Woody	Fleshy	Spring
*Asclepias syriaca*	Apocynaceae	Native	Herbaceous	Dry	Spring
*Asclepias tuberosa*	Apocynaceae	Native	Herbaceous	Dry	Summer
Cardamine pensylvanica^*^	Brassicaceae	Native	Herbaceous	Dry	Spring
*Diervilla lonicera*	Caprifoliaceae	Native	Woody	Dry	Summer
Erigeron annuus^*^	Asteraceae	Native	Herbaceous	Dry	Spring
*Eupatorium fistulosum*	Asteraceae	Native	Herbaceous	Dry	Summer
*Ilex verticillata*	Aquifoliaceae	Native	Woody	Fleshy	Spring
*Lobelia cardinalis*	Campanulaceae	Native	Herbaceous	Dry	Summer
*Monarda fistulosa*	Lamiaceae	Native	Herbaceous	Dry	Summer
Oenothera biennis^*^	Onagraceae	Native	Herbaceous	Dry	Summer
Oxalis stricta^*^	Oxalidaceae	Native	Herbaceous	Dry	Spring
*Penstemon digitalis*	Plantaginaceae	Native	Herbaceous	Dry	Summer
Persicaria lapathifolia^*^	Polygonaceae	Native	Herbaceous	Dry	Summer
*Phlox paniculata*	Polemoniaceae	Native	Herbaceous	Dry	Summer
*Polygonatum biflorum*	Ruscaceae	Native	Herbaceous	Fleshy	Spring
*Rhododendron periclymenoides*	Ericaceae	Native	Woody	Dry	Spring
*Solidago juncea*	Asteraceae	Native	Herbaceous	Dry	Summer
*Viburnum recognitum*	Adoxaceae	Native	Woody	Fleshy	Spring
*Viola sagittata*	Violaceae	Native	Herbaceous	Dry	Spring
*Viola sororia*	Violaceae	Native	Herbaceous	Dry	Spring
*Alliaria petiolata*	Brassicaceae	Non-Native	Herbaceous	Dry	Spring
*Buddleja davidii*	Scrophulariaceae	Non-Native	Woody	Dry	Summer
*Carduus nutans*	Asteraceae	Non-Native	Herbaceous	Dry	Spring
*Chelidonium majus*	Papaveraceae	Non-Native	Herbaceous	Dry	Spring
*Consolida ajacis*	Ranunculaceae	Non-Native	Herbaceous	Dry	Summer
Coronilla varia^*^	Fabaceae	Non-Native	Herbaceous	Dry	Summer
*Datura stramonium*	Solanaceae	Non-Native	Herbaceous	Dry	Summer
*Hesperis matronalis*	Brassicaceae	Non-Native	Herbaceous	Dry	Spring
Lamium purpureum^*^	Lamiaceae	Non-Native	Herbaceous	Dry	Spring
*Ligustrum obtusifolium*	Oleaceae	Non-Native	Woody	Fleshy	Spring
*Lonicera japonica*	Caprifoliaceae	Non-Native	Woody	Fleshy	Spring
Lysimachia nummularia^*^	Myrsinaceae	Non-Native	Herbaceous	Dry	Spring
*Lythrum salicaria*	Lythraceae	Non-Native	Herbaceous	Dry	Summer
*Solanum dulcamara*	Solanaceae	Non-Native	Herbaceous	Fleshy	Spring
*Viburnum acerifolium*	Adoxaceae	Non-Native	Woody	Fleshy	Summer

**Notes.**

*Species with flowering periods longer than 5 months excluded in some analyses.

We converted each specimen’s collection date to an integer reflecting the ordered day within a year. We classified each species with regard to native status, growth form (woody *vs* herbaceous), fruit type (dry *vs* fleshy), and blooming season (spring *vs* summer) (Table 1). We identified native status and growth form using The PLANTS Database of the United States Department of Agriculture, Natural Resources Conservation Service (USDA, NRCS, 2021b). Species were classified as native if they occured in an area before European settlement, or non-native if they were introduced to an area by humans (USDA NRCS, 2021a). For each species, we established the blooming time as spring or summer based on the season in which the largest proportion of blooming period months fell, as per Rhoads, Block & Aniśko (2007). We removed all records that had collection dates falling outside of a species-specific range of two standard deviations from the mean, keeping the middle 95% of observations (Bolmgren & Lönnberg, 2005; RI Bertin, 2021, pers. comm.).

### Analyses

In order to predict changes in flowering date in response to changes in temperature over time, we created three linear mixed-effects models. Each model used a different climate variable as the predictor: average yearly temperature (YearTAVG), average winter temperature (WinterTAVG), and average spring onset temperature (SpringTAVG). In all models, species was treated as a random effect to account for the variability of flowering dates between species. We calculated confidence intervals for the change in predicted flowering date based on a unit change in temperature as indicated by the slopes of each model. In instances of non-normal residuals or heteroscedasticity, we used a non-parametric bootstrap method to calculate the 95% confidence intervals. Confidence intervals that do not include the value of 0 indicate a significant change in flowering date per 1 °C change in temperature, with positive slopes indicating later flowering dates and negative slopes indicating earlier flowering dates. We report all significant slopes generated by the model.

To determine if native status, growth type, fruit type, and blooming season had statistically significant effects on flowering date, we ran four additional mixed-effects models to include each of the binary variables and their interaction with temperature, continuing to use species as a random effect. For each of the three temperature periods (YearTAVG, WinterTAVG, and SpringTAVG), we analyzed how collection date differed across the following categorical variables: native status (native or non-native), growth habit (woody or herbaceous), fruit type (dry or fleshy), and seasonality (spring - or summer - blooming). Confidence intervals for the effect of native status, growth type, fruit type, seasonality, as well as their interactions were calculated. Again, in instances of heteroscedasticity or where the residuals were non-normal, non-parametric bootstrapping was used to calculate the 95% confidence intervals. Confidence intervals that do not include the value of 0 indicate a significant difference in the change in flowering date between native status, growth type, fruit type, and spring- or summer-blooming. Significant values for the coefficient of the interaction term (*β*_temp*variable) within the model indicate that temperature has a significantly different effect on flowering phenology between the categorical variables. To account for the possibility that the inclusion of some species with considerably longer blooming times in our analyses may obscure underlying patterns that characterize the majority of species that have well-defined, shorter flowering periods, we re-ran the above analyses excluding species with flowering periods longer than 5 months (Table 1). All statistical analyses were performed using R (version 1.2.5033) statistical computing language through RStudio (RStudio Team, 2020), with the following packages: mosaic, readr, tidyr, dplyr, lme4, ggpubr, and rstatix (R Core Team, 2020).

## Results

**Figure 1:**
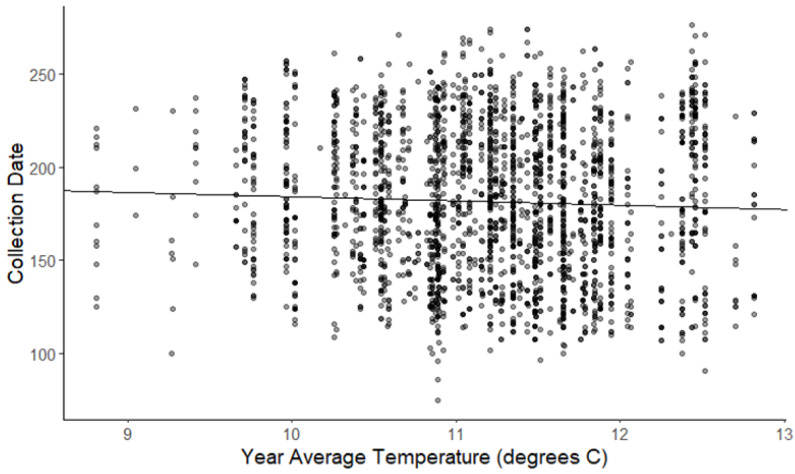
Plot of collection date *vs* yearly average temperatures for all species. The darkness of each point reflects the number of observations with the same value. (*β* = −2.26, 95% CI [−3.27 to −1.26], significant).

**Figure 2:**
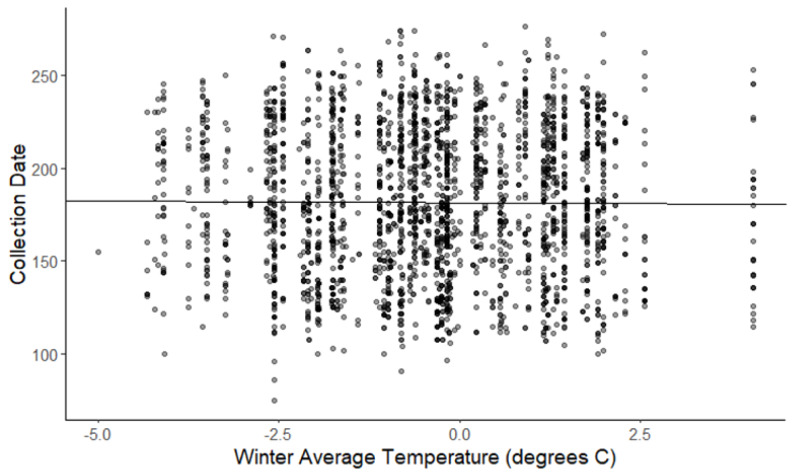
Plot of collection date *vs* winter average temperatures for all species. The darkness of each point reflects the number of observations with the same value (*β* = −0.162, 95% CI [−0.59 to 0.38], n.s).

**Figure 3:**
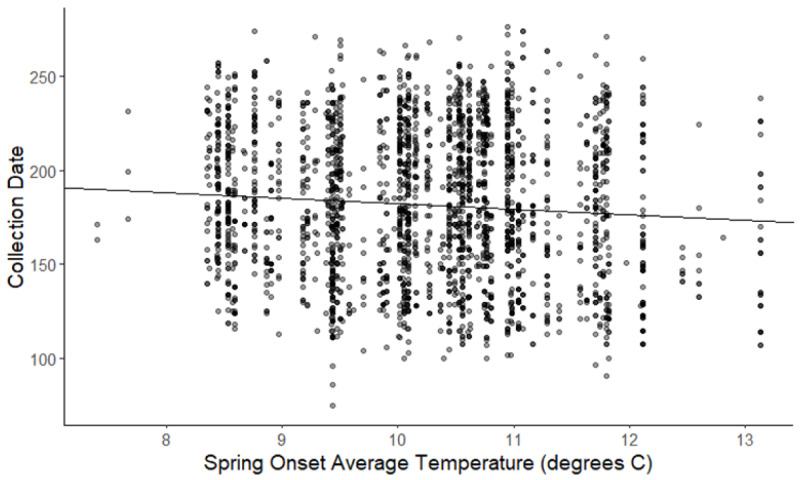
Plot of collection date *vs* average onset spring temperatures for all species. The darkness of each point reflects the number of observations with the same value (*β* = −2.93, 95% CI [−3.62 to −2.27], significant).

**Figure 4:**
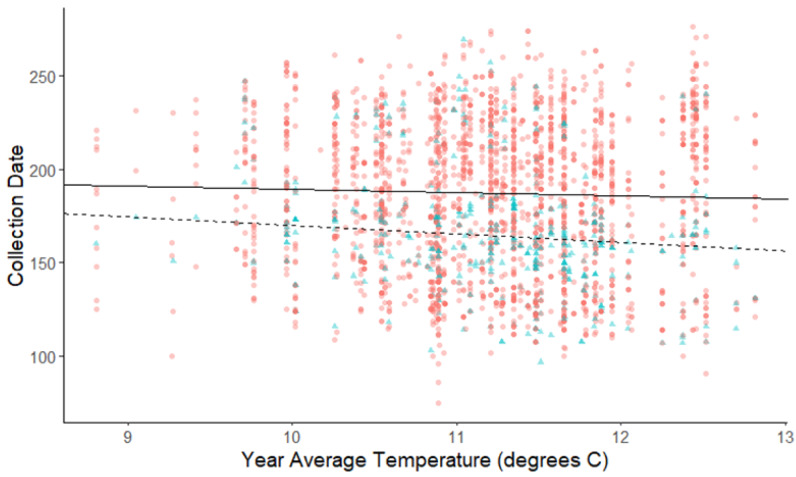
Plot of collection date *vs* average yearly temperatures for herbaceous plants and woody plants species. The red dots and solid line are for herbaceous plant species, and the blue triangles and dashed line are for woody plant species. The darkness of each point reflects the number of observations with the same value. Refer to Table 2 for test statistics.

The linear mixed-effects regressions for all species with individual species as the random effect were significant for annual and spring temperatures, but not winter temperatures, as indicated by whether or not the 95% confidence intervals included the value of 0. These analyses revealed that from 1884–2015, plants in this study on average bloomed approximately 2.26 days earlier per 1 °C increase in the yearly average temperature (*β* = −2.26, 95% CI [−3.27 to −1.26], Fig. 1). Winter temperatures did not significantly influence flowering phenology (*β* = −0.16, 95% CI [−0.59 to 0.38], Fig. 2), while plants flowered approximately 2.93 days earlier per 1 °C increase in the spring onset average temperatures (*β* = −2.93, 95% CI [−3.62 to −2.27], Fig. 3). When we excluded species with extended blooming periods beyond five months the effect was slightly stronger for yearly average (*β* = −2.72, 95% CI [−3.96 to −1.74]), and spring onset temperatures (*β* = −3.16, 95% CI [−3.83 to −2.38]) and remained non-significant for winter temperatures (*β* = 0.09, 95% CI [−0.39 to 0.55]). There was no significant difference in the effect of temperature on flowering phenology between native and non-native species for any of the three temperature periods (Table 2). The comparison between woody and herbaceous growth plant species showed a significant difference in flowering phenology only in response to the yearly average temperature, with woody species (slope = −4.53 d/° C) flowering 2.85 days earlier than herbaceous species (slope = −1.68 d/° C) with increasing temperatures (95% CI [−4.919 to −0.822]) (Table 2, Fig. 4).

**Table 2:** Results from mixed-model regressions by native status, growth type, fruit type, and blooming season. Interaction terms and 95% CIs listed in bold indicate significance. Slopes for individual categories in each regression reported in the text are calculated based on which category is named in the interaction term. For the category named in the interaction term the slope is the sum of *β*_temp and the *β*_temp*category value. For the other category in the comparison, the slope is *β*_temp.

**Native *vs* Non-native**					
Parameter	*β*_temp	*β*_nonnat	*β*_temp*nonat	95% Confidence Interval	
				Lower-95	Upper-95
YearTAVG	−2.367	−6.873	0.324	−1.769	2.518
WinterTAVG	0.185	5.346	−0.270	−1.328	0.843
SpringTAVG	−2.925	−3.585	−0.002	−1.46	1.599
**Woody *vs* Herbaceous**					
Parameter	*β*_temp	*β*_woody	*β*_temp*woody	95% Confidence Interval	
				Lower-95	Upper-95
YearTAVG	−1.681	9.341	**−2.852**	**−4.919**	**−0.822**
WinterTAVG	−0.191	−22.893	0.240	−0.812	1.247
SpringTAVG	−2.749	−14.494	−0.776	−2.013	0.632
**Fleshy *vs* Dry Fruit**					
Parameter	*β*_temp	*β*_fleshy	*β*_temp*fleshy	95% Confidence Interval	
				Lower-95	Upper-95
YearTAVG	−2.133	−15.777	−0.448	−3.41	1.904
WinterTAVG	−0.149	−20.981	−0.025	−1.243	1.207
SpringTAVG	−3.062	−27.938	0.704	−0.875	2.596
**Spring *vs* Summer**					
Parameter	*β*_temp	*β*_summer	*β*_temp*summer	95% Confidence Interval	
				Lower-95	Upper-95
YearTAVG	−3.284	19.196	**2.27**	**0.485**	**4.662**
WinterTAVG	−0.324	44.866	0.369	−0.702	1.239
SpringTAVG	−3.208	38.363	0.611	−0.573	2.006

**Figure 5:**
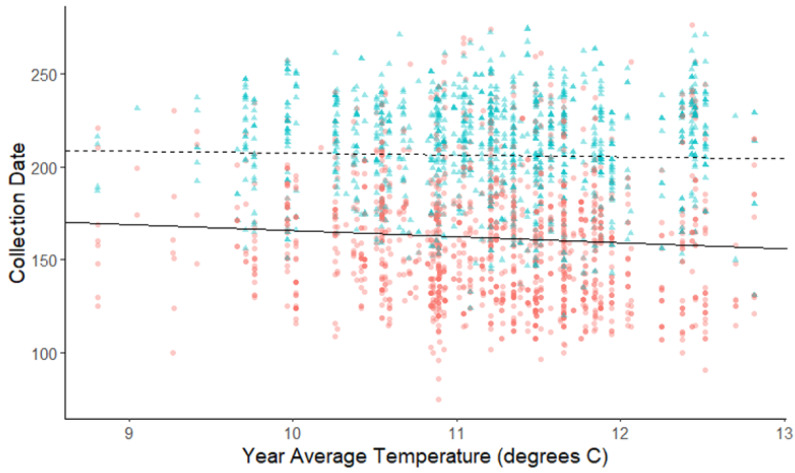
Plot of collection date *vs* average yearly temperatures for spring-blooming and summer-blooming species. The red dots and solid line are for spring-blooming species, and the blue triangles and dashed line are for summer blooming species. The darkness of each point reflects the number of observations with the same value. Refer to Table 2 for test statistics.

For the comparison of fruit types, there was no significant difference in flowering phenology in response to warming annual, winter, or spring temperatures (Table 2). The comparison between spring and summer blooming species was significant only for yearly average temperatures (Table 2, Fig. 5). Spring blooming species exhibited a significantly greater response to warming (slope = −3.284 d/° C) than summer blooming species (slope = −1.014 d/° C), with the difference in slopes revealing that spring blooming species flowered 2.27 days earlier per 1 °C increase in yearly average temperature than summer blooming species (95% CI [0.485 to 4.662]). The exclusion of species with extended flower periods did not influence any of the results of the categorical analyses in Table 2.

## Discussion

We have shown that in eastern Pennsylvania flowering phenology has significantly advanced in response to rising temperatures. The species in this study bloomed on average approximately 2.26 days earlier per 1 °C increase in the yearly average temperature from 1884–2015 (Fig. 1). This is consistent with other similar studies showing plants flowering 2–6 days earlier (Primack et al., 2004; Miller-Rushing et al., 2006; Robbirt et al., 2011; Anderson et al., 2012; Panchen et al., 2012; Calinger, Queenborough & Curtis, 2013; Hart et al., 2014; Bertin, 2015; Davis et al., 2015; Park & Schwartz, 2015; Bertin et al., 2017; Pearson, 2019; Williams et al., 2021; Bloom, O’Leary & Riginos, 2022; Yang et al., 2022). The plants in this study are on the low end of this range for earlier flowering with warming. One possible explanation for this is that much of the area of study remained as undeveloped agricultural land through the early 1990s (Lehigh Valley Planning Commission, 2022) and none of the specimens used in this study were collected from large urban or industrial centers, perhaps in contrast to other areas in which this type of study have been conducted. This may have reduced any heat island effect thereby amplifying warming trends and is known to influence flower phenology (Neil & Wu, 2006; Zipper et al., 2016). Another potential issue is the general decline in herbarium collection rates in more recent decades which could result in an under estimate of phenological response to warming in more recent years. This is the case for a few of the species in our study, which we attempted to address through the use of temperature rather than year as an independent variable in analyses. However, it is possible that this resulted in an underestimation of the of the phenological response to warming in more recent years. Also consistent with prior studies showing early flowering with warming for a number of species, is that not all species flowered earlier and some actually flowered later (Cook, Wolkovich & Parmesan, 2012). Furthermore, differences in the plant species between studies of this sort could explain variation in results due to possible phylogenetic constraints on the phenological responses to warming.

To further explore this variation among species and to shed additional light on the possible mechanistic bases of changes in flowering time with climate change, we also considered the influence of seasonal temperatures within a year and a number of plant functional characteristics that could potentially influence the response of flowering time to warming. In our analysis of the influence of seasonal temperatures on flowering time across all species considered here, we found that warmer spring temperatures were a strong driver of phenological response with plants on average flowering 2.93 days earlier per 1 °C (Fig. 3), which was greater than the response to increases in annual temperature (Fig. 1). This makes sense given the conventional thinking that earlier spring warming would initiate the termination of dormancy, germination, and leaf and flower emergence; and is consistent with prior studies that examined this (Primack et al., 2004; Miller-Rushing et al., 2006; Robbirt et al., 2011; Calinger, Queenborough & Curtis, 2013; Davis et al., 2015; Bertin et al., 2017; Park et al., 2018). However, the question remains why some plant species appeared not to respond to warming temperatures.

The lack of response of some species to warming average may actually be driven by warmer winter temperatures which can delay dormancy or chilling requirements for germination *via* vernalization, which in turn could delay spring events such as flowering or leafing out in some species (Hart et al., 2014; Rubin & Friedman, 2018; Cook, Wolkovich & Parmesan, 2012). This is consistent with our finding that increasing winter temperatures were not significantly related to flowering phenology, and the lack of a negative slope in this relationship, suggests that some species may actually be flowering later in response to warming winter temperatures (Fig. 2). This supports the suggestion by Cook, Wolkovich & Parmesan (2012) that species that appear not to respond to warming average annual temperatures seen in this and other studies may actually be responding to warming, but the opposing effects of winter and spring warming cancel each other out. Sampling error and limited sample sizes may also be a possible explanation for the varied response among species.

We expected the native status of species to influence phenological responses to temporal warming because of the potentially greater phenotypic plasticity of non-native species (Richards et al., 2006; Davidson, Jennions & Nicotra, 2011), but we found no difference between native and non-native species in the relationship between flowering time and rising annual and seasonal temperatures (Table 2). Other studies that considered this found similar or mixed results (Holle, Wei & Nickerson, 2010; Bertin, 2015; Bertin et al., 2017), with only one other that found non-native species to flower earlier in response to rising temperatures (Calinger, Queenborough & Curtis, 2013).

There are a number of possible explanations why we did not see non-native species flowering earlier in response to warming than natives. First, not all introduced species are invasive, and whether or not a plant is invasive may be a more important determinant of plasticity than native status. This however is difficult to discern as most studies of plasticity in non-native plants focus on those that are invasive. Since only species with specimens that span the time range over which warming has occurred and that the rate of introduction has increased exponentially during the time of this study (Lehan et al., 2013), the proportion of non-native species that are invasive may have been under represented in this and similar studies. In fact, only five of the non-native species used in this study, *Alliaria petiolata, Datura stramonium, Hesperis matronalis, Lonicera japonica* and *Lythrum salicaria*, and *Lonicera japonica* (ital) (Table 1) are designated as invasive (USDA NRCS, 2021a). If invasive non-natives exhibit greater phenotypic plasticity than those that are not invasive, the underrepresentation of invasive species in our study could explain the lack of response to warming of the non-native species seen here and in other studies. Also, most studies of invasive plants assess plasticity characters such as reproductive output, growth rate, and photosynthesis (Richards et al., 2006), and plants with greater plasticity may also respond to warming by investing more in early vegetative growth. Finally, adaptive selection on phenological advance also plays a significant role in response to warming (Anderson et al., 2012), potentially equalizing differences in the response of native and non-native plants attributed to plasticity. If we are to better understand how phenological changes may influence biological invasion with climate warming, we will need to advance our understanding of the underlying mechanisms.

We found that the relationship between flowering time and warming was significantly stronger in woody plants than that in herbaceous plants, contradicting our own hypothesis. We are unaware of other studies that directly make this same comparison. There is some evidence that in woody species phenology tends to be cued by photoperiod rather than temperature; but this tendency varies within particular growth forms, and with latitude, flowering season, and other factors (Saikkonen et al., 2012; Zohner et al., 2016; Flynn & Wolkovich, 2018). Thus, to better predict the consequences of climate warming on flowering phenology, direct comparisons among species with different growth forms and life histories are needed, as is a more comprehensive understanding of the relative effects of temperature and day length on phenology across species. Our finding of no differences in phenological response between species with dry or fleshy fruits (Table 2) is also in conflict with our own hypotheses and prior work (Bolmgren & Lönnberg, 2005). This may be due to the low number of fleshy fruited species in our study, or the possibility that fruit type may or may not be related to flowering time or other functional characteristics.

We found that spring-blooming species show a greater advance in flowering phenology than summer-blooming species. Most past studies found the same (Calinger, Queenborough & Curtis, 2013; Bertin, 2015; Bertin et al., 2017; Pearson, 2019), with Park et al. (2018) finding the opposite. The greater advance in flowering time with warming of spring blooming species can simply be explained by the earlier cue of warming on phenological processes. Also, selection on spring blooming plants to flower earlier to decrease competition for pollinators, particularly for male function, is typically balanced by the risk of late winter damage (Niesenbaum, 1992; Austen & Weis, 2016). As that risk decreases with climate warming, selection to flower earlier should become stronger (Calinger, Queenborough & Curtis, 2013). Pearson (2019) demonstrated this effect in a much warmer climate where spring blooming species exhibited a stronger phenological response to warmer spring temperatures than species that bloom later in the season; and that significantly warmer summer temperatures actually delayed flowering in summer and autumn flowering species. Additionally, Pearson (2019) found that the phenological advance in spring flowering was dampened in years with high levels of spring precipitation independent of temperature.

## Conclusion

It is clear from our study and others like it that climate change is influencing flowering time in ways that could negatively impact community level ecological processes. However, it also clear that the phenological responses to warming varies greatly among species due to the multiple factors and mechanisms at play. These include differential responses to winter and spring warming, and different functional characteristics among those species. Future work should focus on these possible mechanisms through both historical and experimental studies that take into account the complexities of seasonal differences in warming, and multiple factors that likely contribute to the variation in the response of individual species and how they might interact or be confounded with each other. Consideration of other factors related climate change such as seasonal precipitation, snow melt, the frequency and intensity extreme weather events, and comparisons among sites with greatly different climates will further enhance our understanding of how climate change influences flowering phenology (Lavoie & Lachance, 2006; Anderson et al., 2012; Peterson, 2016; Flynn & Wolkovich, 2018; Hufft et al., 2018; Dorji et al., 2020). Additionally, analysis of species specific factors that cue flowering phenology (Davis et al., 2015), the consideration of the influence of urban heat island effects related to urban and suburban development (Roetzer et al., 2000; Ziska et al., 2003; Lavoie & Lachance, 2006), potential interactions among the plant characteristics that we considered, as well as taking into account any phylogenetic signal in flowering response to warming could help to make sense of observed differences in the phenological response to warming.

##  Supplemental Information

10.7717/peerj.15188/supp-1 Supplemental Information 1 Climate Data

10.7717/peerj.15188/supp-2 Supplemental Information 2 Analyses R Code

10.7717/peerj.15188/supp-3 Supplemental Information 3 Phenology Data

## Additional Information and Declarations

### Data Availability

The following information was supplied regarding data availability: The raw data is available in the Supplemental Files.
